# Impact of non-standardized reporting on reproducibility, usability, and integration in nasopharyngeal metagenomic research: a systematic review

**DOI:** 10.3389/fmicb.2026.1707004

**Published:** 2026-02-19

**Authors:** Monica L. Bustos, Kuncheng Song, Hayden N. Brochu, Qimin Zhang, Lakshmanan K. Iyer, Crystal R. Icenhour

**Affiliations:** Labcorp, Burlington, NC, United States

**Keywords:** nasopharyngeal microbiome, reproducibility, usability, integration, standardized reporting, methodological transparency, accessible metadata, data integration

## Abstract

**Introduction:**

The nasopharyngeal microbiome plays a critical role in respiratory health and disease and is a major focus of metagenomic research. However, inconsistent reporting practices across studies limit reproducibility, dataset usability, and cross-study integration, thereby reducing the overall scientific value of publicly available nasopharyn.

**Methods:**

A systematic review was conducted to evaluate the impact of non-standardized reporting on reproducibility, usability, and integration of nasopharyngeal metagenomic datasets. A total of 988 studies were screened, and 227 manuscripts met predefined inclusion and exclusion criteria for full-text review. Methodological reproducibility, metadata completeness, and compatibility between reported laboratory methods and deposited datasets were assessed. Reproducible datasets were further analyzed to evaluate the interchangeability of nasopharyngeal aspirates and nasopharyngeal swabs.

**Results:**

Only 78 studies (34%) contained methods sections sufficient for reproducibility, and of these, 33 studies (15%) provided analytically sufficient metadata to support secondary analysis. Mismatches between reported laboratory methods and deposited datasets were identified in 4% of studies. These deficiencies were primarily attributed to incomplete methodological reporting, inaccessible or insufficient metadata, and incompatible file formats. Comparative analysis of reproducible datasets demonstrated significant differences in microbial profiles between nasopharyngeal aspirates and nasopharyngeal swabs, confirming that these specimen types are not interchangeable within a study.

**Discussion:**

The findings demonstrate that inadequate reporting standards substantially impair the reproducibility, reuse, and integration of nasopharyngeal metagenomic data. The observed methodological and metadata inconsistencies limit the reliability of downstream analyses and cross-study comparisons. Standardized reporting guidelines are urgently needed to improve transparency, ensure reproducibility, and enhance the integrative potential of nasopharyngeal microbiome research. Adoption of comprehensive and consistent reporting practices would significantly strengthen the scientific rigor and utility of metagenomic studies in this field.

## Introduction

The nasopharyngeal microbiome, a diverse population of microorganisms residing in the upper respiratory tract, plays a crucial role in maintaining respiratory health and influencing disease susceptibility (Gao et al., 2014; Esposito and Principi, 2018; Wypych et al., 2019). As sequencing technologies advance and metagenomic approaches evolve, the nasopharyngeal microbiome has become a key focus of research, particularly in the context of infectious diseases, chronic conditions like asthma and sinusitis, and the impact of the microbiome on immune system modulation (Belkaid and Hand, 2014; Durack and Lynch, 2019). Despite the generation of vast nasopharyngeal microbiome datasets, challenges related to data reproducibility, usability, and integration persist, impeding expansion of metagenomic research (Kumar et al., 2024; Ma et al., 2024). These issues are exacerbated by the lack of standardized methodological documentation and reproducible workflows across studies, which hinders the validation of findings, the reuse of data in future research, and the integration of datasets from diverse sources or studies (Vangay et al., 2021). The lack of reproducible data in published metagenomic studies has significant and wide-ranging implications, including delayed or ineffective medical and public health applications, ineffective interventions, and wasted resources through duplicated research efforts (Vangay et al., 2021; Schloss, 2018). Given the diversity of sequencing approaches available for microbiome characterization, it is important to clarify the methodological scope considered within this review.

Microbiome research encompasses several distinct laboratory methodologies, each with unique analytical implications. While approaches such as metagenomic and metatranscriptomic sequencing enable broad genomic and transcriptional characterization, these methods remain less practical for nasopharyngeal studies due to the inherently low microbial biomass and high abundance of host nucleic acids in this specimen type. Although host depletion strategies have been developed to mitigate this challenge, they introduce additional wet-lab complexity, increase cost, and may inadvertently distort native microbial compositions through selective nucleic acid removal (Shi et al., 2022; Wang et al., 2025). In contrast, 16S rRNA gene sequencing is not subject to these host or cost constraints and remains the most widely adopted approach in nasopharyngeal microbiome research. Internal transcribed spacer (ITS) sequencing, another amplicon-based method targeting fungal communities, was excluded from this review, as fungal infections are comparatively rare in the nasopharyngeal environment and are rarely studied for respiratory infections (Avendaño Carvajal and Perret Pérez, 2020). Although combined 16S + ITS sequencing would ideally capture both bacterial and fungal diversity, such dual analyses are uncommon due to the additional financial and technical demands they impose. Eventually, we focused on the most widely used and accessible technology, 16S rRNA gene sequencing. This decision is central to the study’s aim: if the most common and foundational methods in the field are not reproducible, it cannot be assumed that more complex or resource-intensive methods will perform better. By establishing reproducibility benchmarks within the dominant methodological framework, this review provides a realistic foundation for improving standardization and transparency across nasopharyngeal microbiome research. By defining this methodological scope, the present review provides a uniform framework for evaluating reproducibility and methodological transparency within the current nasopharyngeal microbiome literature.

Within this defined context, however, major challenges remain regarding data reproducibility, usability, and integration, which continue to limit the broader applicability of nasopharyngeal microbiome research. Inconsistent methodological practices in metagenomic data generation and analysis manifest in multiple ways within nasopharyngeal microbiome studies, including inconsistent descriptions of sampling methods, variations in sequencing techniques, and inadequate metadata, such as demographic or clinical information (Findley et al., 2016). These inconsistencies complicate efforts to reproduce results, making it difficult for independent researchers to verify findings or conduct similar studies using the same methodologies (Qian et al., 2020). Furthermore, without transparent reporting of methods and data processing workflows, researchers struggle to understand how datasets were generated, hindering effective data reuse and cross-study comparisons (Schloss, 2018). Integration of nasopharyngeal microbiome datasets from different studies is similarly hindered by these reporting challenges, limiting the capacity to draw broader conclusions about microbial diversity and its clinical relevance across populations and disease states (Vangay et al., 2021). Although several reporting frameworks have been introduced to address these challenges, they often lack the specificity needed for nasopharyngeal and other low-biomass respiratory sources.

Despite the availability of broad standardization frameworks such as the Minimum Information about a Metagenomic Experiment (MIMS) (Ten Hoopen et al., 2017, STrengthening the Reporting Of Microbiome Studies) (STORMS) (Mirzayi et al., 2020) metadata templates, significant implementation gaps persist within nasopharyngeal metagenomic research. Existing frameworks emphasize general metadata capture and minimal sequencing descriptors but often overlook the domain-specific nuances required for respiratory sampling, such as specimen collection method (nasopharyngeal swab versus aspirate), extraction biases in low-biomass samples, and alignment between reported protocols and deposited datasets (Amos et al., 2020). Moreover, while several prior reviews have assessed microbiome data reproducibility across diverse anatomic sites, none have directly evaluated the reproducibility and usability of publicly available nasopharyngeal datasets or quantified the extent of methodological misalignment between published methods and archived sequence data (Amos et al., 2020). Consequently, there remains no standardized mechanism to evaluate the reporting fidelity of nasopharyngeal sequencing studies, nor a unified benchmark for determining dataset readiness for integration into large-scale surveillance initiatives. Addressing these deficiencies represents a critical step toward establishing transparent, reproducible, and interoperable metagenomic research practices in respiratory microbiome studies.

Building upon these gaps, this systematic review sought to evaluate publicly available nasopharyngeal microbiome publications to assess the reproducibility, usability, and integration readiness of their datasets. Characterizing the healthy nasopharyngeal microbiome remains an emerging and complex undertaking, requiring a broad and diverse dataset to ensure analytical robustness and reproducibility. To support the standardization of a nasopharyngeal bioinformatic resource for genomic surveillance of respiratory pathogen outbreaks, this systematic review evaluated publicly available nasopharyngeal microbiome publications. The objectives of this study were to assess the extent to which published nasopharyngeal microbiome studies provide sufficient methodological detail for independent reproducibility, to evaluate the completeness and accessibility of associated metadata determining dataset usability, and to examine the degree of methodological consistency and compatibility necessary for cross-study data integration. We hypothesized that inconsistent methodological documentation and insufficient metadata completeness substantially limit the reproducibility, usability, and integration potential of nasopharyngeal microbiome datasets, thereby reducing their overall value for secondary analysis and large-scale comparative research. Given the distinct methodologies used for each sampling technique, the data were analyzed to determine whether both sample types yielded comparable microbiome profiles. This report aims to highlight critical gaps in standardization, encourage methodological transparency, and provide recommendations to enhance reproducibility and data integration in future nasopharyngeal microbiome research. To evaluate the potential methodological impact of sampling variation, this review included reanalysis of sequence data from a subset of studies that provided sufficient methodological detail and accessible raw data to enable independent validation. Specifically, two studies meeting all reproducibility criteria were reanalyzed to assess whether nasopharyngeal aspirates (NPA) and nasopharyngeal swabs (NPS) the two most common sampling techniques identified in the systematic review, yield comparable microbial community profiles. This comparison directly addresses a frequent methodological inconsistency observed across the reviewed literature and provides an applied example of how reproducibility enables secondary data utilization. By defining these objectives, and incorporating a reproducibility-based reanalysis component, the study establishes a structured framework for identifying key methodological barriers and evaluating practical solutions that can enhance transparency and standardization in nasopharyngeal microbiome research.

## Methods

### Data sources and search strategy

**Figure 1:**
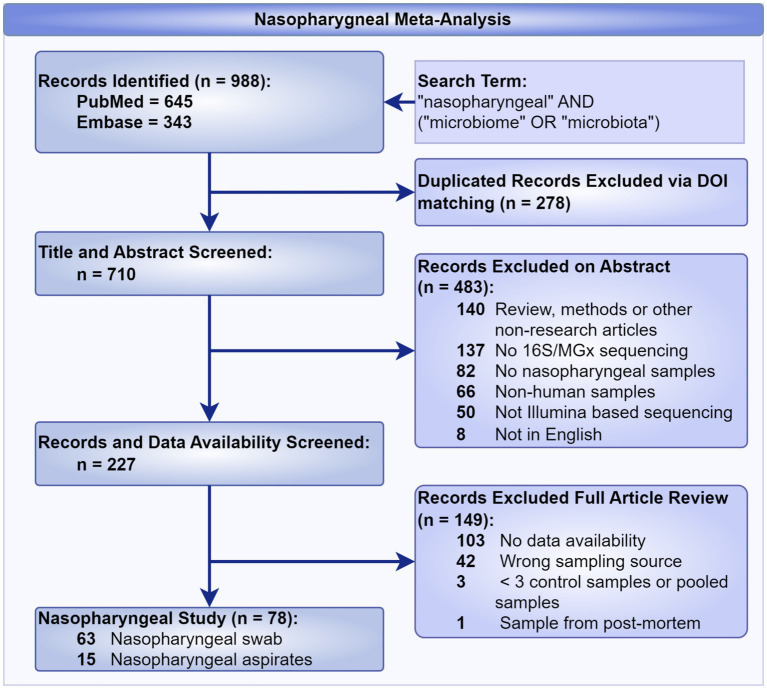
Preferred reporting items for systematic review for the curation of a healthy nasopharyngeal bioinformatic library.

We conducted a systematic search of the PubMed^1^ and Embase^2^ databases with the primary goal of curating publicly accessible nasopharyngeal microbiome publications. The complete Boolean search string used for both databases was (“nasopharyngeal”) AND (“microbiome” OR “microbiota”). No Medical Subject Headings (MeSH) terms were applied in PubMed in order to cast the widest net possible and ensure inclusion of all relevant studies regardless of indexing status. The review followed the Preferred Reporting Items for Systematic Reviews and Meta-Analyses (PRISMA) 2020 guidelines (Page et al., 2021). Although the review was not prospectively registered in a public database such as PROSPERO or the Open Science Framework (OSF), the full PRISMA 2020 checklist was completed and is provided as Supplemental File 1. The decision not to register was based on the study’s methodological scope, which focuses on evaluating reproducibility and standardization practices rather than clinical or interventional outcomes. Within this framework, this review also assessed the reproducibility and replicability of healthy nasopharyngeal whole-genome datasets, emphasizing the impact of non-standardized reporting on their usability and integration in future research. The search strategy employed (Figure 1), with the search restricted to studies published up to May 18, 2024. The initial query returned 988 records, which were first deduplicated to remove overlapping studies between databases. The remaining unique records were then subjected to a title and abstract screening by two independent reviewers (MB and KS). This screening process applied strict eligibility criteria to ensure manuscripts represented novel research studies, employed appropriate next-generation sequencing techniques, utilized nasopharyngeal sampling methods, analyzed human subjects, were available in English, and used Illumina sequencing platforms. Studies that did not meet these criteria were excluded. When eligibility was unclear or reviewers disagreed, a third reviewer (CI) resolved the discrepancy. Publications passing initial screening underwent full-text review to confirm adherence to all inclusion and exclusion criteria. The screening process is summarized in Figure 1. Of the 227 studies that met the initial inclusion criteria, 78 were ultimately selected for dataset download and further reprocessing through our standardized bioinformatics pipeline. This meta-analysis curation process was also included in our independent study by Song et al. (2025), which provides a deeper analysis of the nasopharyngeal microbiome to detail the creation of an analytical framework that allows for the translation of nasopharyngeal microbiome data into clinically actionable results. These studies were selected based on the availability and quality of accompanying metadata, which was then evaluated using several key criteria: clear differentiation between control and disease states, accurate alignment of reported methods with raw data files, metadata completeness, and the identification of baseline data in longitudinal designs. These requirements ensured that selected datasets were structured to support robust and reproducible downstream analysis.

During the meta-analysis conducted for the curation of publicly available healthy nasopharyngeal microbiome data, a significant portion of the initial records were excluded due to issues with data accessibility and methodological reporting. Out of 227 manuscripts selected for full article review, 149 were excluded, primarily because of inaccessible data or discrepancies within the methods section. These limitations in data availability and reporting highlighted critical challenges in ensuring reproducibility and reliability, which directly influenced the inclusion and exclusion criteria for this review. This finding led the reviewers to recognize potential broader issues with methodological reproducibility, prompting a secondary review focused on assessing the replicability of the methodologies employed. Of the 227 records, 78 articles were identified as having nasopharyngeal sources, which were subsequently selected for a more rigorous evaluation of their methodology. The criteria used in this process are outlined below.

### Inclusion and exclusion criteria

Inclusion Criteria (studies were included in the meta-analysis if they met the following criteria):

1. Provided metagenomic sequencing data in a publicly accessible format.
2. Focused on nasopharyngeal specimen collection.
3. Reported relevant outcomes, such as microbial diversity or specific taxonomic abundances.

Exclusion Criteria (studies were excluded based on the following criteria):

1. Lack of data availability (including original datasets, publicly available datasets, and Illumina-based 16S/metagenomic studies).
2. Absence of clearly defined nasopharyngeal specimen collection.
3. Fewer than three unique subjects (data could not be pooled across sources or individuals).
4. Inclusion of post-mortem samples.

### Data extraction, reproducibility, and quality assessment

**Figure 2:**
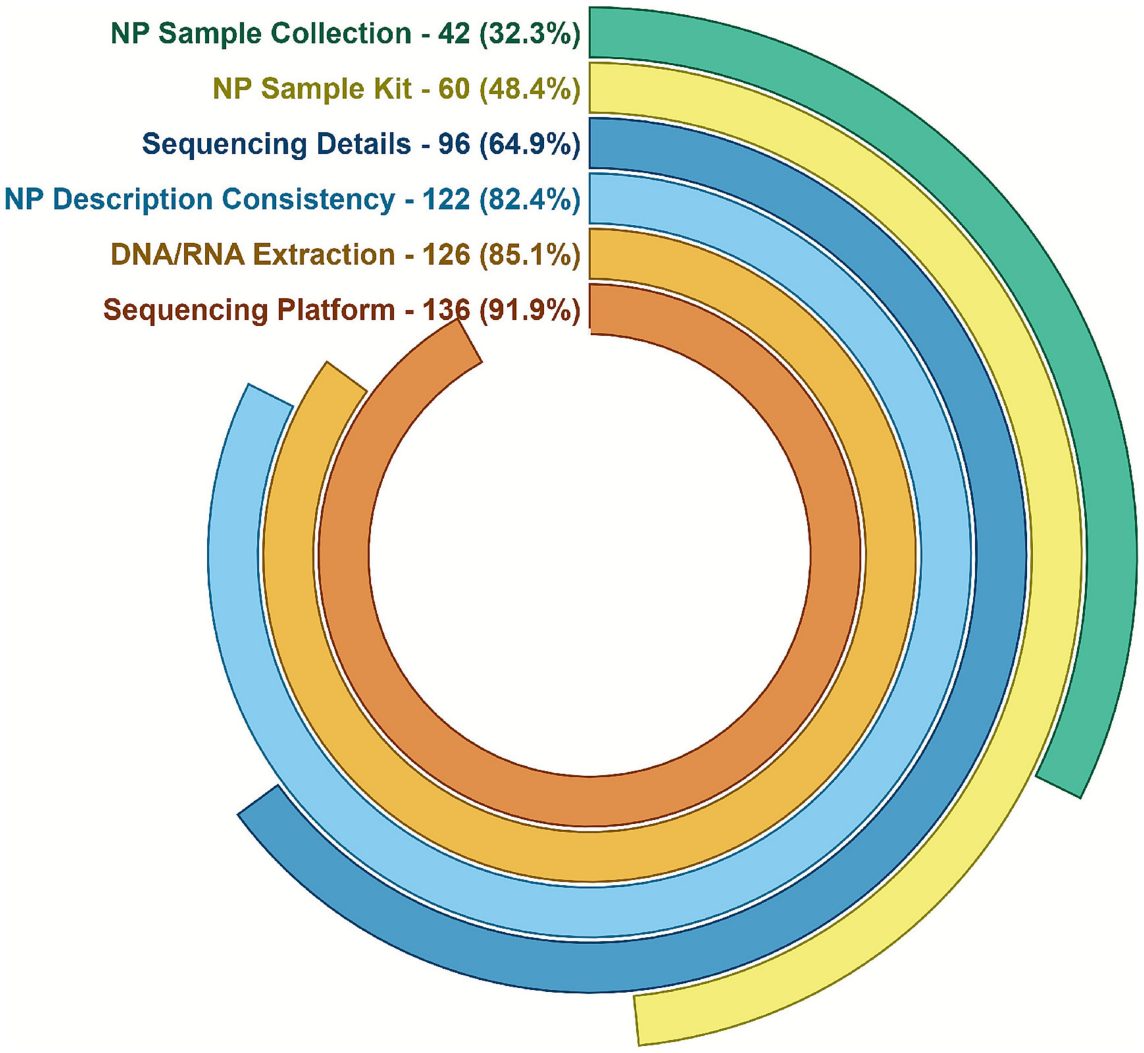
Methodological reproducibility assessment of 148 nasopharyngeal microbiome studies. Studies were evaluated for specific reproducibility barriers including, from outer ring to inner ring: (1) Lack of sampling details (insufficient description of collection method, storage, or timing), (2) sampling inconsistencies (conflicting information within methods section), (3) incomplete sequencing details (missing manufacturer/catalog information), (4) inconsistency in the use of the nasopharyngeal source terminology indicating or referring to other sources, (5) unreported or missing sequencing procedures (missing DNA/RNA extraction kit or protocol), and (6) lack of methodological transparency (sequencing platform information and manufacturer). Percentages and absolute numbers are shown for each category. Categories are not mutually exclusive; studies could exhibit multiple deficiencies.

Data extraction was performed by two independent reviewers (MB and KS) using a standardized Excel-based data collection form designed to capture reproducibility parameters across all included studies. The extraction process was conducted in duplicate to ensure accuracy and completeness. Any discrepancies between the two reviewers were resolved through discussion and consensus, with CI serving as a third independent adjudicator when agreement could not be reached. The data extraction form included predefined fields corresponding to each methodological parameter listed in Figure 2, ensuring consistency and reproducibility in the review process itself.

The methodological variables extracted for each study included: (1) sampling method and description of nasopharyngeal (NP) specimen collection, (2) sample collection kit with manufacturer, (3) specimen storage conditions, (4) DNA or RNA extraction kit and protocol with name and manufacturer, (5) sequencing platform and system with manufacturer, (6) sequencing chemistry and parameters including targeted region (e.g., V3–V4), primer sequences, read length, and sequencing depth, (7) bioinformatics pipeline and software versions used for analysis, and (8) disease or population of interest. In total, 148 publications were evaluated using these parameters to assess methodological transparency and reproducibility.

To determine whether a study was classified as reproducible, a minimum-threshold approach was applied, and conformed with MIMC recommendations. Studies were considered reproducible if they provided sufficient methodological detail to enable independent replication without relying on secondary citations. This included: (1) a clear description of NP sample collection methods; (2) the specific collection kit and manufacturer; (3) comprehensive sequencing details, including primers, targeted 16S rRNA region, sequencing chemistry, read length, and depth; (4) consistent use of the NP source designation throughout the text, avoiding interchangeable or ambiguous terminology such as “nasal,” “oropharyngeal,” or “nasopharyngeal”; (5) the DNA/RNA extraction kit with manufacturer and catalog information; and (6) the sequencing platform and system with manufacturer. To qualify as reproducible, all parameters had to be explicitly reported within the manuscript or had to correctly align to the cited publications.

Reliance on citation-based methods, where authors reference another study’s methods without providing sufficient detail, was not accepted as adequate documentation. In nearly all such instances, indirect citation chains failed to provide enough methodological overlap to accurately replicate the original workflow and were thus categorized as non-reproducible. Methodological inconsistencies were further classified as within-study or between-study. Within-study inconsistencies referred to internal contradictions, such as mismatched sequencing platforms between text and raw data, mislabeled sample types (e.g., NPS vs. NPA), or conflicting descriptions of collection procedures. Between-study inconsistencies reflected expected methodological heterogeneity across studies, such as differing extraction kits, primers, or storage methods. While between-study variation represents normal methodological diversity, frequent within-study inconsistencies exacerbate cross-study comparability challenges and contribute significantly to reproducibility limitations within the nasopharyngeal microbiome field.

Study quality was evaluated through the same reproducibility framework described above, emphasizing methodological completeness, data accessibility, and internal consistency. Traditional indicators such as study design and sample size were considered contextually but were not used as exclusion criteria. Instead, the primary quality determinants were the presence of sufficient methodological detail to meet reproducibility thresholds and the absence of within-study inconsistencies. Selected datasets meeting these criteria are detailed in the following section.

### DADA2 processing, quality control, and taxonomic analysis

Raw 16S V4 rRNA gene sequencing data were obtained from NCBI BioProject PRJNA997934 (nasopharyngeal swabs, NPS) and 16S V3V4 rRNA gene sequencing data from PRJNA275918 (nasopharyngeal aspirates, NPA) using fasterq-dump from the NCBI SRA Toolkit v3.1.0 (NCBI SRA Toolkit, 2025). To match the infant-only cohort in the NPA dataset (age <1 year), NPS samples were filtered accordingly. Only samples labeled as healthy were retained from both datasets.

Of the 78 studies that met inclusion criteria, two datasets were selected for comparative reanalysis to evaluate reproducibility and the potential impact of sample type on nasopharyngeal microbiome results. These datasets were chosen because they met stringent selection criteria that ensured comparability and data quality: (1) infant populations (<1 year of age), (2) healthy subjects only, (3) high sequencing depth with publicly accessible raw data, and (4) complete associated metadata. The two BioProjects included PRJNA997934 (Odendaal et al., 2024), representing nasopharyngeal swab (NPS) samples, and PRJNA275918 (Teo et al., 2015), representing nasopharyngeal aspirate (NPA) samples. These studies were selected as representative, high-quality examples within the broader dataset because they employed similar 16S rRNA gene sequencing methodologies and provided sufficient methodological transparency to permit reproducible reanalysis.

Read processing was performed in R v4.1.1 (R: a language and environment for statistical computing, 2025). For the NPA dataset (V3V4 region), reads were reoriented when necessary. All reads were then trimmed using the filterAndTrim function in DADA2 v1.22.0 (Callahan et al., 2016). Read lengths were 2 × 250 bp (NPS) and 2 × 150 bp (NPA), with primers excluded. An in-house R function optimized trimming based on quality profiles, primer presence, and expected insert length (~256 bp). Trim parameters were set to trimLeft = c(10,10) and trimRight = c(40,189) for NPS, and c(20,19) for NPA. Quality-filtered reads underwent standard DADA2 processing for denoising, merging, and generation of amplicon sequence variants (ASVs). Singletons and ASVs <350 bp were removed. Taxonomic classification was performed using the SILVA v138.2 database (Lin and Peddada, 2020) with the DADA2 assignTaxonomy (minBoot = 80) and addSpecies functions. ASVs lacking at least family-level classification were excluded. Remaining ASVs were aggregated to their lowest assigned taxonomic rank to generate the count matrix. To mitigate batch effects, taxa commonly associated with laboratory or environmental contamination (e.g., kit/reagents origin microbes known collectively as “kitome,” marine, or soil microbes) were removed. Only samples with ≥5,000 reads were retained, yielding the final aggregated count matrix for downstream analysis.

To ensure comparability, only infant (<1 year) and healthy subject samples were retained, matching the cohort characteristics between datasets. This age-matched, healthy-only design allowed assessment of how differences in sample collection method (swab vs. aspirate) could influence downstream community composition and reproducibility across otherwise comparable studies.

### Alpha diversity, dissimilarity, and differential abundance analyses

Alpha diversity was calculated using the Shannon index via the diversity function (vegan v2.6–2) (McMurdie and Holmes, 2013). Rarefaction was performed across thresholds from 5,000 to 100,000 reads (in 5,000-read increments) using the rrarefy function, repeated five times per threshold. Median values were used per sample, and Wilcoxon rank-sum tests assessed diversity differences between groups. Community composition differences were evaluated using Bray–Curtis dissimilarity and visualized via multidimensional scaling (MDS) with cmdscale. Statistical significance of clustering was tested using PERMANOVA (adonis2) with 999 permutations. Differential abundance (DA) analysis between NPS and NPA groups was conducted in R v4.4.3 (Findley et al., 2016) using ancombc2 (ANCOM-BC v2.8.1) (Lin and Peddada, 2020) with input formatted via phyloseq v1.50.0 (McMurdie and Holmes, 2013). Taxa with FDR-adjusted *p* < 0.05 were considered significant. Sensitivity to pseudo-count selection was noted. Taxa with positive or negative log2 fold-change (L2FC) were classified as enriched or depleted, respectively.

### Statistical analysis

False discovery rate (FDR) correction was applied via the Benjamini-Hochberg method for all multiple hypothesis testing. Co-exclusive microbial detections were evaluated using Pearson’s Chi-squared test.

### Data availability

All datasets included in this meta-analysis are publicly available from NCBI SRA, and links to the datasets can be found in the Supplementary materials section.

## Results

In this systematic review, we initially screened 988 manuscripts by abstract and title, ultimately selecting 227 for full-text review. Upon further examination, 148 manuscripts contained appropriate qualifying nasopharyngeal sources for curating publicly available healthy nasopharyngeal bioinformatics data. However, only 78 articles contained reproducible methods sections, as assessed using the screening criteria outlined in Figure 2. The secondary review evaluated the impact of non-standardized reporting on the reproducibility, usability, and integration of metagenomic datasets in subsequent research. The studies were selected based on criteria that mandated the use of metagenomic sequencing to characterize the nasopharyngeal microbiome, with a specific focus on challenges related to reproducibility, replicability, clarity of terminology, usability, and the integration of datasets across studies. The results are structured around three principal themes: reproducibility, usability, and the integration of nasopharyngeal microbiome datasets.

### Reproducibility

Our evaluation of methodology across the 148 selected studies revealed significant reporting gaps that hindered reproducibility and comparability of nasopharyngeal microbiome research. These gaps were particularly evident in three key areas: sampling methods, sequencing and extraction methods, and overall methodological transparency. Many studies lacked sufficient detail regarding nasopharyngeal sampling procedures, with variations in collection protocols and storage conditions, making it difficult to replicate studies or compare results across cohorts.

To assess data acceptability, we reviewed the methodology of 148 studies (Figure 2). A significant proportion lacked sufficient detail in their nasopharyngeal sampling procedures (*n* = 102, 68.9%) or exhibited inconsistencies in sample collection protocols (*n* = 26, 17.6%). These inconsistencies included variations in the method of collection, storage conditions, and the specific sources from which samples were collected. Furthermore, 88 studies (59.5%) did not report essential information regarding the collection kits used, such as the type of swab collection kit or the transport medium, leaving critical gaps in the methodology. These shortcomings posed substantial challenges for replicating studies or comparing results across cohorts. For instance, variations in the type of swab used (e.g., nasal swabs versus nasopharyngeal swabs) and discrepancies in sample preservation methods led to differences in microbial profiles, making it difficult to reproduce findings and draw consistent conclusions. These issues underscore the need for more standardized and transparent reporting in microbiome research to ensure the reliability and comparability of results across studies.

Additionally, many studies failed to specify critical details about extraction kits, sequencing procedures, and associated variables such as read depth, contributing to discrepancies in microbiome characterization. Among the 148 studies reviewed for methodological transparency, 22 studies (14.9%) did not specify the extraction method or kit for isolating DNA/RNA from nasopharyngeal samples. This lack of information is concerning, as variations in extraction protocols can significantly impact the quality and composition of the extracted DNA/RNA, thereby influencing subsequent analyses. In addition, 52 studies (35.1%) failed to disclose key sequencing procedures, such as the sequencing format used during 16S sequencing (i.e., PCR primers and hypervariable region(s) targeted). Sequencing format is a critical parameter that can affect the accuracy and resolution of taxonomic and functional precision of the detected microbes. Without this information, assessing the reliability of reported microbial profiles and performing cross-study comparisons becomes difficult. These omissions highlight the importance of detailed and standardized reporting in sequencing methodologies, as even minor differences in extraction and sequencing techniques can lead to substantial discrepancies in microbiome data.

Finally, many studies lacked transparency regarding control samples, sequencing platform-specific biases, and software versions, all of which are essential for ensuring the reproducibility of findings. Twelve studies (8.1%) failed to report critical methodological details essential for reproducibility and comparability of results. These studies omitted information about control samples, sequencing platform-specific biases, and/or software versions used in their analyses. Such omissions are particularly concerning given the diversity of sequencing platforms currently available, each with its own set of potential biases that can significantly influence microbiome study outcomes. Furthermore, the use of different taxonomic databases across studies added another layer of complexity. Variations in database selection, combined with inconsistent thresholds for taxonomic classification, contributed substantially to discrepancies in reported microbiome compositions. These inconsistencies create barriers to direct comparison between studies and complicate result interpretation. The lack of transparency regarding these methodological choices highlights the urgent need for standardization in nasopharyngeal microbiome research to facilitate reliable cross-study comparisons.

Of the 148 studies selected for methodology review, we identified 78 with sufficiently reproducible methods sections, which we subsequently selected for dataset download and metadata analysis. Despite this careful selection, the lack of standardized sampling methods, sequencing protocols, and bioinformatics workflows significantly impeded reproducibility across nasopharyngeal microbiome studies. Inconsistencies in methodological reporting directly contributed to variations in microbial composition across studies, making independent replication difficult or impossible in many cases. These methodological discrepancies underscore the critical need for greater standardization in both the design and reporting of microbiome studies to ensure reliable, reproducible findings that can advance the field.

### Usability

**Figure 3:**
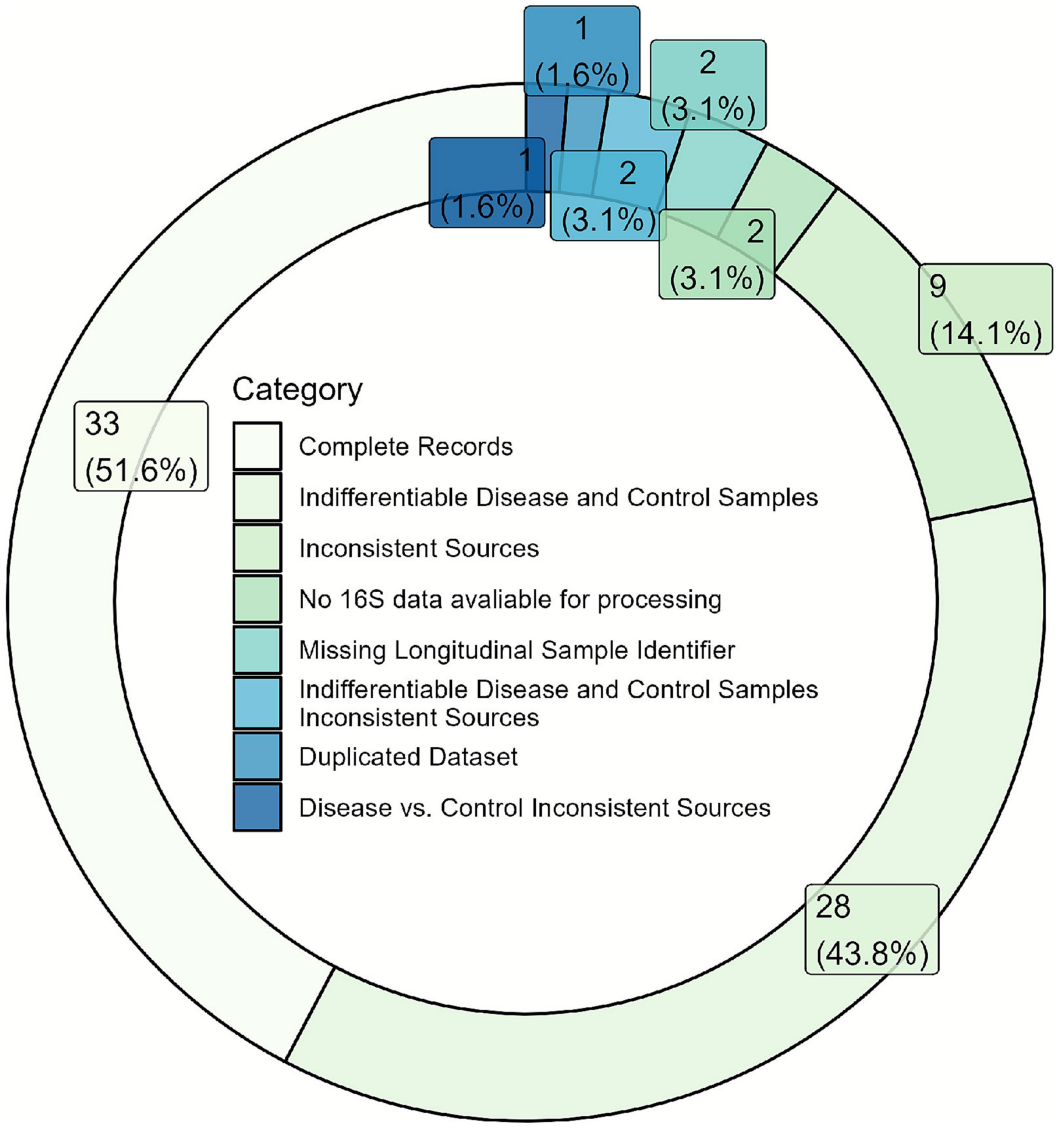
Metadata quality assessment of 78 studies with reproducible methods sections. Studies were evaluated for metadata completeness and accuracy by comparing deposited metadata files against published methods. Categories include: (1) Complete and reproducible data, (2) Disease status not reported (inability to distinguish control from case samples in metadata), (3) Sources conflicting with methods (mismatch between sample types reported in manuscript versus metadata files), (4) No 16S data available under the NCBI BioProject, (5) No baseline identifier for longitudinal data (time series studies lacking time-point zero or pre-intervention identification), (6) Files not marked with disease/control and show different sources conflicting with the methods section, (7) Datasets duplicated with another study, (8) Labeled sources are inconsistent and different from methods section details. Only studies with publicly available datasets were assessed (*n* = 78). Percentages and absolute counts are shown for each deficiency type.

Of the 148 manuscripts reviewed for methodology, we identified only 78 with sufficiently reproducible methods sections for dataset download and metadata analysis. While all 78 studies provided publicly available data, significant usability issues hindered their practical application in further research. As Figure 3 illustrates, 45 studies (57.7%) exhibited usability challenges, primarily related to difficulties in accessing, interpreting, and utilizing the metadata associated with the original datasets. A frequent issue was the inadequate reporting of participant characteristics, such as age, gender, disease status, and antibiotic usage, all essential for contextualizing microbiome data in clinical or ecological studies. Specifically, two studies (3.1%) did not identify sources in multi-source studies, while nine studies (14.1%) listed sources that conflicted with their methods sections. Furthermore, 28 studies (43.8%) omitted disease status information, precluding differentiation between control and experimental groups, and two studies (3.1%) lacked baseline identifiers for longitudinal analyses. The absence of these critical clinical and demographic variables limited the ability to assess the relevance of the microbiome findings or to determine whether the results could be generalized to other populations or contexts. Consequently, despite data availability, these usability issues substantially reduced the value of the datasets for secondary analyses and replication efforts. Insufficient metadata and difficulties in data access significantly limited the usability of nasopharyngeal microbiome datasets. The lack of transparency in data sharing further restricted the potential for secondary analysis and wider dissemination of findings.

### Integration

We identified integration issues in 2 studies (3.1%), primarily stemming from misidentification of sequencing file types. These studies reported using one sequencing platform in their methods sections (e.g., Illumina) but instead employed a different platform (e.g., 454), resulting in unexpected file formats. This discrepancy creates significant technical barriers because bioinformatic pipelines are typically designed for specific file types (e.g., FASTQ or BAM) and cannot process misidentified file types without substantial modification. Aside from technical challenges, these inconsistencies between reported and actual sequencing platforms compromise data integrity and reproducibility. Such integration barriers effectively prevent large-scale meta-analyses and comparative studies that could otherwise provide comprehensive insights into the role of the nasopharyngeal microbiome in health and disease.

### Sampling discrepancies for the nasopharyngeal source

Although commonly occurring in nasopharyngeal studies, the alternating method of nasopharyngeal sampling, whether through aspirate (NPA) or swab (NPS), may influence the microbial profiles detected by sequencing.

**Figure 4:**
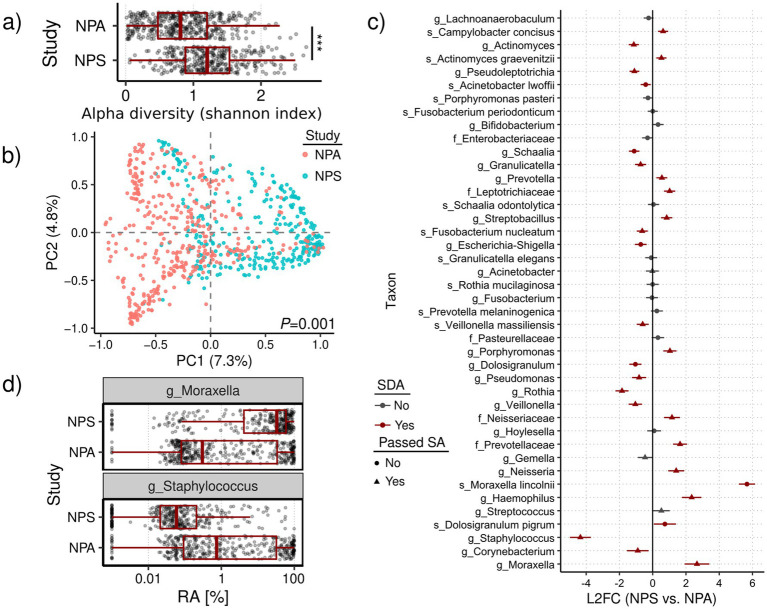
Comparison of nasopharyngeal swab (NPS) and aspirate (NPA) microbiomes. **(a)** Alpha diversity computed using the Shannon index compared across studies with boxplots overlayed by jittered sample data points. **(b)** Multidimensional scaling (MDS) of pairwise Bray-Curtsi dissimilarities (BCDs) with samples colored by study. In the MDS plot, the first two dimensions are shown, each with variation explained by the dimensions in parentheses. Statistical significance of sample grouping based on BCDs was determined using PERMANOVA, with *p*-value shown on bottom-right. **(c)** Comparison of taxa (*y*-axis) log-2 fold changes (L2FCs, axis) between NPS and NPA samples. Each taxon is colored based on whether it was SDA (red) or not (gray), and the shape of the data point indicates whether the taxon passed sensitivity analysis (SA) (triangles) or not (circles). ANCOM-BC2 employs SA by evaluating whether results change if different pseudo-counts are used to replace zeros in the dataset (if so, then the taxon fails SA). Horizontal bars for each data point represent the 95% confidence interval of the L2FC measurement computed using its standard error. Positioning of the data points/bars to the right of 0 indicates an enrichment of taxa in NPS samples relative to NPA samples, while those to the left of 0 are depleted. Taxa are ordered by mean relative abundance (most abundant at the bottom of plot). **(d)** Boxplots of two highly dissimilar taxa found in **(c)**, showing relative abundances (RAs) stratified by study (log_10_ axis transformation).

Raw 16S rRNA gene sequencing data (Table 1) were processed using DADA2 to generate amplicon sequence variants (ASVs) aggregated taxonomically to reduce sparsity. Given that the studies were conducted in different laboratories using different protocols, we applied stringent background filtering to remove potential contaminants. Of 1,240 initial taxa, 608 (~49%) were identified as background. Despite their number, these taxa were rare, with median relative abundances (RAs) of 0.3% (NPA) and 0.7% (NPS) (Figure 4a). Their removal reduced batch effects by eliminating taxa with disproportionately high prevalence in one dataset, such as *f_Bacillaceae* (95% NPA vs. 7% NPS) and *g_Ralstonia* (0.3% NPA vs. 58% NPS) (Figure 4b). After filtering and retaining only samples with ≥5,000 reads, we obtained a final dataset of 632 taxa across 317 NPS and 391 NPA samples.

**Table 1:** Summary of NPS and NPA datasets analyzed.

Study	NCBI BioProject	Sample type	Age range	Total samples	Age-matched, healthy, & filtered samples
Odendaal et al. (2024)	PRJNA997934	NPS	All	2,799	317
Teo et al. (2015)	PRJNA275918	NPA	2–12 m/o	913	391

We first assessed overall diversity and composition. Alpha diversity was significantly higher in NPS samples compared to NPA (Figure 4a; Wilcoxon rank-sum test, *W* = 87,737, *p* < 0.0001), a pattern consistent across multiple rarefaction thresholds. Beta diversity analysis using Bray–Curtis dissimilarity showed significant differences in community structure between the two sampling methods (Figure 4c; PERMANOVA, *p* < 0.001).

To identify differentially abundant taxa, we applied ANCOM-BC2 to the full dataset. Of the 632 taxa, 42 met prevalence thresholds for analysis, and 27 (64%) were significantly differentially abundant (SDA; *p* < 0.05) (Figure 4c). Among these, 21 passed the sensitivity analysis. Notable differences included enrichment of *g_Moraxella* (L2FC = 2.7) and *g_Haemophilus* (L2FC = 2.3) in NPS samples, and *g_Staphylococcus* (L2FC = -4.3) in NPA samples. The species *s_Moraxella lincolnii* had the largest L2FC (5.7) but was excluded during sensitivity analysis, likely due to insufficient resolution in the V4 region. Further analysis revealed higher *g_Moraxella* and lower *g_Staphylococcus* relative abundance in NPS samples (Figure 4d). We also observed a strong co-exclusion pattern between *g_Moraxella* and *g_Staphylococcus*: in 70% of samples (494 of 706), only one was detected above 1% relative abundance (Table 2; Pearson’s Chi-squared test, *X^2^* = 143.81, df = 3, *p* < 0.0001).

**Table 2:** Comparison of *g_Moraxella* and *g_Staphylococcus* detection between NPA and NPS samples, using a detection threshold of 1% relative abundance (RA).

*g_Moraxella* RA > 1%?	*g_Staphylococcus* RA > 1%?	Number of NPA samples	Number of NPS samples
No	No	90	52
No	Yes	133	12
Yes	No	121	228
Yes	Yes	45	25

Together, these findings demonstrate that the choice of sampling method significantly influences the diversity and taxonomic composition of the nasopharyngeal microbiome as recovered by sequencing.

## Discussion

Our analysis identified substantial gaps in reproducibility, usability, and integration, demonstrating how inconsistent methodological documentation and a lack of workflow standardization severely limit the scientific value and reusability of metagenomic datasets, undermining their potential to contribute to robust and replicable scientific discoveries.

### Reproducibility challenges in metagenomic studies

Reproducibility, a cornerstone of scientific rigor, is severely hampered by inadequate reporting methods in nasopharyngeal metagenomic studies. In our study, only 78 out of 148 (52.7%) of the analyzed studies met the reproducibility criteria for methods sections, suggesting that many studies lack the necessary detail for other researchers to replicate their findings. This gap in reporting includes insufficient information on critical steps, such as DNA/RNA extraction found to be missing in 22 (14.9%) studies, sequencing protocols missing in 12 (8.1%) studies, source designation missing in 26 (17.5%) studies, and sample collection missing in 106 (71.6%) studies, which are essential to ensure that results can be independently verified. The lack of detail in these areas creates uncertainty around reported findings, limits trust in conclusions drawn from the data, and makes it challenging for researchers to build on prior studies. Improving reproducibility through standardized and comprehensive reporting would enable more reliable comparisons across studies, advancing our understanding of microbial ecology in the nasopharynx.

### Usability constraints due to incomplete metadata

Inconsistent reporting also limits the usability of data for new research contexts, with metadata availability emerging as a significant challenge. Of the 78 studies with reproducible methods sections, only 33 (42.3%) had usable metadata in public datasets. Metadata—information that describes the context, conditions, and parameters of an experimental dataset—is essential for researchers looking to reanalyze or apply data beyond the scope of the original study. In the absence of comprehensive and accessible metadata, the usability of data is compromised, as researchers lack the contextual information necessary to conduct accurate analyses or to interpret results meaningfully. To maximize the potential of metagenomic data, we recommend that journals and repositories enforce standardized metadata submission that includes, at minimum, clear subject demographics, health status, sampling protocols, and experimental conditions.

Furthermore, our findings revealed that 3.1% of nasopharyngeal metagenomic studies had mismatched laboratory methods, where the sequencing methods reported did not correspond with the actual data formats in public repositories. This misalignment further complicates usability, as it reduces the reliability of the data and raises fundamental questions about the accuracy and validity of study conclusions. Standardized protocols for both reporting methods and metadata alignment are critical for ensuring that publicly available datasets are usable and accurately reflect the experimental conditions under which they were generated.

### NPS versus NPA sampling for the nasopharyngeal source

Comparative analysis of nasopharyngeal swab (NPS) and aspirate (NPA) samples revealed significant differences in microbial diversity and community composition, with profound implications for reproducibility in nasopharyngeal microbiome research. We identified *g_Moraxella*, *g_Haemophilus*, and *g_Staphylococcus* as key taxa differentially abundant between the two sampling methods, suggesting that sampling technique plays a critical role in shaping observed microbiome profiles.

One of the most striking findings was the markedly higher abundance of *g_Staphylococcus* in NPA samples (L2FC = −4.3), indicating more than a 16-fold increase relative to NPS samples. This substantial enrichment likely reflects the increased capture of oral-associated microbes during aspiration-based collection. Unlike swabbing, which typically targets the posterior nasopharynx with minimal disturbance to surrounding areas, aspirates may collect a broader range of microorganisms from the upper respiratory tract, including the oropharyngeal space.

*g_Moraxella* and *g_Haemophilus* were significantly enriched in NPS samples, and both taxa are considered common and clinically relevant colonizers of the nasopharynx. Their higher relative abundance in swab-collected samples may suggest that NPS more effectively targets the proper nasopharyngeal niche, while aspirates may dilute this signal with oral contaminants. Similarly, although less pronounced, *g_Rothia* also showed a trend toward differential abundance, further supporting the influence of sampling method on community structure.

These findings underscore the critical importance of standardized sampling protocols in nasopharyngeal microbiome research. The apparent compositional disparities observed between NPA and NPS methods emphasize the need for meticulous documentation of collection procedures. Without a consistent methodology, cross-study comparisons may be confounded by sampling artifacts, potentially leading to misleading conclusions in research and clinical contexts. As microbiome-based diagnostics and interventions gain traction, avoiding methodological bias through standardized collection protocols and transparent reporting is essential to ensure accuracy and reproducibility.

### Acknowledgement of confounding factors

While we controlled for age by comparing only infant cohorts and for health status by including only healthy subjects, several potential confounders remain that may influence the observed differences between NPA and NPS microbiomes. Even within infant populations, subtle developmental changes in the nasopharyngeal environment can occur over weeks to months, potentially altering microbial profiles (De Steenhuijsen Piters et al., 2022). The definition of “healthy” also varies between studies, some excluded recent infections or antibiotic exposure, whereas others did not explicitly state exclusion criteria. Additionally, environmental and geographic differences between the two study populations may have shaped baseline microbiome composition. Technical variability related to swab depth, angle, and anatomical targeting could introduce sampling bias, as some swabbing techniques target specific subregions of the nasopharynx more precisely than others. The original study aims and collection protocols may also influence rigor, handling, and storage practices. Temporal factors such as the year of collection and differences in sequencing chemistry or reagent lots can further contribute to variability, despite efforts to standardize bioinformatic processing.

To minimize these potential biases, extensive quality control filtering, consistent data processing, and removal of known contaminant taxa were applied. Nonetheless, certain factors, such as subtle anatomical targeting differences and unreported environmental exposures, could not be fully controlled. Therefore, the observed differences between NPA and NPS datasets may reflect both the influence of sampling method and residual cohort-level confounders. Paired sampling, in which both NPA and NPS specimens are collected from the same subjects under controlled conditions, would be necessary to definitively isolate the effect of the collection method alone.

These results highlight the need for within-study standardization and precise documentation of collection techniques rather than a universal preference for one sampling method over another. The observed differences in taxonomic composition underscore the challenges of integrating data across studies that use distinct nasopharyngeal collection approaches. Future work employing paired or longitudinal sampling across diverse populations will be essential to establish the reproducibility and interchangeability of nasopharyngeal sampling methods in microbiome research.

### Implications for data integration across studies

Integration of data from multiple studies is essential for large-scale analyses that drive a comprehensive understanding of metagenomics, but the inconsistent reporting practices we identified severely hinder this process. Data integration requires that methodologies, metadata, and laboratory procedures align across studies for reliable aggregation and comparative analyses. The discrepancies in reproducibility, metadata usability, and method compatibility identified in this study highlight the challenges in combining datasets for more expansive research opportunities. Misaligned or inadequately reported methods introduce variability and may lead to spurious results when datasets are integrated. For example, combining NPS and NPA datasets without accounting for their inherent microbial profiling differences could yield artificial clusters in meta-analyses that reflect methodology rather than biology. Adopting standardized methodological protocols that clearly document sample collection, sequencing, and data processing methods is essential to support meaningful data integration necessary for larger initiatives, such as population-scale microbiome-disease association studies and respiratory pathogen surveillance.

### Recommendations and future directions

Our findings underscore the pressing need for standardized methodological frameworks and workflow transparency in nasopharyngeal metagenomics, which could potentially have broader applications in other metagenomic fields. Consistent criteria for reporting methodological details, metadata, and data alignment practices would benefit reproducibility, usability, and integration across studies. While open data sharing is a cornerstone of reproducibility, it must be conducted in accordance with institutional review board (IRB) oversight and ethical data-sharing principles. Particular care should be taken when working with data derived from vulnerable populations, such as pediatric or immunocompromised subjects. Full transparency in data provenance must be accompanied by safeguards for privacy, consent, and secure data management, ensuring that open access does not compromise participant protection (Ma et al., 2018).

To address the reproducibility challenges identified in our review, we propose a comprehensive framework for standardized methodological design and reproducible workflow documentation in metagenomic research. It is important to acknowledge that there is currently no universally accepted “gold standard” for microbiome sequencing methods. Unlike clinical assays, microbiome analyses lack absolute reference materials that define the most accurate or superior approach. Consequently, this study does not aim to determine which method is “best,” but rather to emphasize that any chosen method must be reported transparently and reproducibly so that results can be critically evaluated and replicated.

Metagenomic sequencing is an invaluable tool for studying the complex microbial communities that inhabit diverse environments, from human microbiomes to soil ecosystems. However, ensuring the reproducibility and replicability of metagenomic research requires detailed and transparent reporting of sequencing data. As a best practice, researchers should provide comprehensive metadata that covers the “how,” “why,” and “where” of data generation, including technical and procedural details ranging from sample collection through bioinformatic analysis protocols. Based on our systematic review findings, we have identified several critical reporting elements that should be considered essential in nasopharyngeal research publications. These elements generally fall into five key areas: (1) sequencing platform and technology, (2) sample processing and DNA extraction, (3) library preparation and sequencing depth, (4) bioinformatics and quality control, and (5) contextual metadata.

Previous literature, including Amos et al. (2020), and other foundational efforts such as the STORMS (Amos et al., 2020) and MIMS frameworks, have provided valuable guidance on metadata standardization and transparent reporting (1). Our findings build upon these initiatives by emphasizing reproducibility-focused adaptations specific to nasopharyngeal microbiome research, particularly for describing sample collection, extraction kits, sequencing chemistries, and bioinformatics reproducibility metrics.

The sequencing platform chosen for metagenomic studies significantly impacts the quality, depth, and data type obtained. Different sequencing platforms come with their advantages and limitations, so understanding these details is essential for reproducibility and for comparing results across studies. Researchers should identify the sequencing platform used, such as Illumina, Oxford Nanopore, PacBio, or Roche 454, and specify the exact model (e.g., Illumina NovaSeq, MiSeq) and the manufacturer. Each platform has inherent biases, read lengths, and error profiles that can influence sequencing outcomes. For example, Illumina platforms generate short, high-accuracy reads, while Oxford Nanopore offers longer reads better suited for genome assembly but with a higher error rate. The sequencing technology should also be specified—whether next-generation (NGS) or third-generation (TGS). As a best practice, mention whether shotgun sequencing (whole-genome) or targeted sequencing (e.g., 16S rRNA) was employed, as these produce different data types and require distinct analytical pipelines.

Describing the DNA extraction protocol used, including the specific kit and any modifications, is crucial. DNA extraction methods can introduce biases in microbial recovery, especially in complex microbial communities. Some methods may preferentially extract DNA from certain taxa or fail to recover others. Additionally, researchers should specify the library preparation kit employed, as different kits can affect the amplification or fragmentation of DNA. Proper documentation ensures that other researchers can replicate the procedure and assess its impact on data quality and microbial profiling.

Sequencing depth, read quality, and quality-control measures directly affect the resolution and reliability of microbiome characterization. Researchers should clearly report quality thresholds, describe filtering criteria, and identify the number of samples excluded for insufficient depth or poor quality. Transparent reporting enables others to determine whether the dataset meets reproducibility standards and facilitates valid cross-study comparisons.

The bioinformatics pipeline used to process sequencing data must also be reported in full detail, including preprocessing steps, reference databases, taxonomic classifiers, and statistical methods. Differences in pipeline parameters can alter taxonomic assignments, functional annotations, and downstream interpretations, underscoring the importance of clear documentation. By extending the STORMS checklist for nasopharyngeal research, we recommend an adapted framework that emphasizes reproducibility of sample methodology, extraction, and analytical transparency.

Beyond technical parameters, contextual metadata, such as study geography, environmental context, collection year, subject age range, and health status, must be clearly defined to allow proper interpretation. For longitudinal or comparative work, temporal metadata should also be reported to control for known microbial drift over time.

In conclusion, while metagenomic sequencing offers unparalleled insights into microbial ecosystems, the ability to replicate and compare findings depends on transparent and complete methodological reporting. Our proposed nasopharyngeal-adapted framework aligns with existing initiatives (MIMS and STORMS) and expands them by prioritizing reproducibility, ethical compliance, and open yet secure data sharing. Encouraging journals and funding agencies to require such documentation will help ensure that reproducibility and accountability become standard practice rather than exceptions.

## Conclusion

In conclusion, our systematic review reveals significant challenges in the reproducibility, usability, and integration of nasopharyngeal metagenomic datasets due to insufficient and inconsistent reporting practices. With only 34.3% of studies meeting reproducibility criteria, 42.36% of those studies providing usable metadata, and 3.1% showing mismatched methods, it is evident that methodological and workflow guidelines are urgently needed to advance the reliability and utility of metagenomic research. Strikingly, only 4.6% (33 accepted of the 710 screened) of publicly available datasets were ultimately suitable for advancing the definition of a healthy nasopharyngeal microbiome, highlighting the substantial scientific opportunity cost of poor reporting practices. Our study also highlights how the nasopharyngeal sampling method significantly impacts microbiome composition. Researchers should exercise caution when interpreting results across studies using different collection techniques and prioritize transparent reporting of methodological details. By implementing the standardized reporting practices we outlined, the field can build a stronger foundation for reproducible, usable, and integrative research. Ultimately, improved standardization will enhance the scientific value and clinical impact of metagenomic studies, accelerating discoveries that refine our understanding of the nasopharyngeal microbiome.

## Data Availability

The original contributions presented in the study are included in the article/Supplementary material, further inquiries can be directed to the corresponding author.

## Footnotes

1
https://pubmed.ncbi.nlm.nih.gov/

2
https://www.embase.com/
